# What Is the Evidence on Natural Recovery Over the Year Following Sports-Related and Non-sports-Related Mild Traumatic Brain Injury: A Scoping Review

**DOI:** 10.3389/fneur.2021.756700

**Published:** 2022-01-05

**Authors:** Morgan Brady, Patria A. Hume, Susan Mahon, Alice Theadom

**Affiliations:** ^1^Traumatic Brain Injury Network (TBIN), Faculty of Health and Environmental Sciences, Auckland University of Technology, Auckland, New Zealand; ^2^Sport Performance Research Institute New Zealand (SPRINZ), Faculty of Health and Environmental Sciences, Auckland University of Technology, Auckland, New Zealand

**Keywords:** traumatic brain injury, concussion, review, recovery, sport

## Abstract

**Background:** Treatment approaches often differ dependent upon whether a person experiences a sports-related or a non-sports-related mild traumatic brain injury. It remains unclear if recovery from these injuries is comparable or unique to context of the injury.

**Objective:** To identify knowledge gaps on self-reported outcomes and trajectories between sports- and non-sports-related mild traumatic brain injuries and how they are assessed in adults.

**Methods:** This scoping review used a systematic search of key electronic databases, including PubMed, SPORTDiscus, Embase, MEDLINE, and CINAHL for articles published in 1937 until March 10, 2021. Articles were included if they were available in English; full text published in a peer-reviewed journal; had a prospective or retrospective study design; reported data on mild TBI cases >16 years of age, and included data from at least two time points on self-reported outcomes within 12 months post-injury. A standardized data extraction spreadsheet was used to determine the participant characteristics, definitions, assessment methods, outcomes, and recovery time frames.

**Results:** Following removal of duplicates, the search strategy elicited 6,974 abstracts. Following abstract review, 174 were retained for full text review. Of the 42 articles that met inclusion criteria, 18 were sports related (15 in the USA and three in Canada) and 24 were general population studies (six in USA, three in Canada, three in Australasia, nine in Europe, two in Taiwan, and one in Morocco). Direct comparison in recovery trajectories between the sport and general population studies was difficult, given notable differences in methodology, definitions, types of outcome measures, and timing of follow-up assessments. Only one article reported on both sports-related and non-sports-related traumatic brain injuries separately at comparable timepoints. This study revealed no differences in recovery time frames or overall symptom burden.

**Discussion:** Whilst there is a clear benefit in researching specific subpopulations in detail, standardized outcome measures and follow-up time frames are needed across contexts to facilitate understanding of similarities and differences between sports- and non-sports-related mild traumatic brain injuries to inform clinical treatment.

## Introduction

A mild traumatic brain injury is defined as a traumatically induced transient disturbance of brain function (1). A concussion is often considered to be a subset of mTBI (2). There is considerable debate as to whether concussion is a useful term, with some advocating the term has no clear definition (3), no pathological meaning (4), and lacks diagnostic precision (4, 5). However, others have argued that the term describes a distinct pathophysiological entity with its own diagnostic and management implications (4). Throughout this paper, only the terms mTBI or sports-related brain injury (SR-TBI) (2, 6) have been used. Both these terms include cases of concussion.

Internationally, the reported age-adjusted incidence rates for the general population for mTBI vary between 59.6 and 811 per 100,000 (7), with approximately 80% of injuries classified as being of mild severity (8). Key challenges in the identification and management of mTBI are that patients can present to a range of services and health care professions (9). Even within countries, service provision can be highly variable (10).

Early recovery advice and gradual return to activity have been found to facilitate recovery (11). In many cases, people recover well in the days to weeks following injury; however, more recently, studies have shown that half of people experiencing mTBI report functional limitations (12) and persistent symptoms, including headaches, fatigue, forgetfulness, poor concentration, and impaired information processing (13). For those with persistent symptoms, interdisciplinary rehabilitation approaches have been found to effectively improve recovery (14).

Of the services providing interdisciplinary rehabilitation interventions, some services specifically focus on management of sports-related injuries, whereas other services treat all clinical presentations. Sports- and non-sports-related kinds of mTBI (e.g., injuries sustained during everyday activities, such as through falls, assaults, vehicle crashes or being hit by or hitting the head against an object) are often talked about, researched, and treated differently. Whilst context-specific research and rehabilitation are important to ensure issues such as managing motivation to return to sport too soon are explored, there is also a need to look across different contexts to advance the field as a whole. For example, what is the best treatment approach for an elite athlete who sustains an mTBI through a vehicle accident, is experiencing psychological trauma, but whose main goal is to return to sport, or the person who sustains a sports-related injury but whose main rehabilitation goal is to return to work and be able to drive?

Some studies have proposed that there are notable differences on the impact of the injury with significantly fewer sports-related injuries showing positive MRI findings in comparison to non-sports-related injuries (15). However, it currently remains unclear if there are differences in the subjective experience of recovery. To provide optimal treatment, it will be important for clinicians to be aware of and address any specific differences between injury contexts.

Existing systematic reviews have explored the experience of prolonged symptoms after mTBI; however, they have focused on particular contexts such as sports-related injuries (16) rather than exploring differences between contexts. Consequently, there is a gap in the existing literature exploring the current evidence based on subjective outcomes following sports-related and non-sports-related mTBI.

## Objective

This scoping review aimed to identify knowledge gaps in self-reported outcomes and trajectories between sports- and non-sports-related kinds of mTBI and how they are assessed in adults.

## Methods

Scoping reviews are used to determine the scope of existing literature on a given topic, clarify key concepts or definitions, examine the way research has been conducted, identify key characteristics, and identify knowledge gaps. To ensure a robust approach to identification of the evidence, a systematic literature search is conducted. As scoping reviews aim to map the existing evidence rather than answer a discrete question (as in the case of a systematic review); formal critical appraisal tools are not incorporated into the approach (17). As the aim of this review was to determine what current research there was that identified self-reported outcomes and trajectories between sports and non-sports-related kinds of mTBI and how they are assessed in adults, we chose a scoping review approach rather than a systematic review. Therefore, the review was not registered with PROSPERO.

To identify relevant literature, a systematic search of key electronic databases was conducted from the start date of database entries until March 10, 2021. Databases included PubMed (1966 onward), SPORTDiscus (1985 onward), Embase (1947 onward), MEDLINE (1946 onward), and CINAHL (1937 onward). The search was conducted using Category 1 terms (“traumatic brain injury” OR “mild tbi” OR mtbi OR concuss^*^ OR brain inj^*^ OR head inj^*^ OR “skull fracture” OR “head trauma” OR “craniocerebral trauma” OR “head impact” OR “craniocerebral injury” OR “brain trauma”) and Category 2 terms (“community participation” OR “physical symptom” OR “cognitive symptom” OR “emotional symptom” OR postconcuss^*^ symptom OR post-concuss^*^ symptom OR “symptom improvement” OR “mood” OR “emotion” OR “clinical outcome” OR “clinical recovery” OR “reintegration”). The use of the search terms “concuss^*^” and “brain inj^*^” captured the relevant MeSH terms, including “brain concussion,” “post-concussion syndrome,” and “brain injuries.” An example search strategy is given in Table 1.

**Table 1:** Search strategy.

**Search by**	**Title/abstract**
Category 1 terms	“traumatic brain injury” OR “mild tbi” OR mtbi OR concuss* OR brain inj* OR head inj* OR “skull fracture” OR “head trauma” OR “craniocerebral trauma” OR “head impact” OR “craniocerebral injury” OR “brain trauma”
	**AND**
Category 2 terms	“community participation” OR “physical symptom” OR “cognitive symptom” OR “emotional symptom” OR postconcuss* symptom OR post-concuss* symptom OR “symptom improvement” OR mood OR emotion OR “clinical outcome” OR “clinical recovery” OR reintegration

To establish some control over heterogeneity of different studies (3), inclusion criteria were established. Papers were included if they met inclusion criteria with the study: available in English; published in a peer-reviewed journal; had full text available; had prospective or retrospective study designs; reported data on participants who had suffered from a mild TBI separately; had participants aged over 16 years or age groups were separated; and included at least one follow-up assessment of self-reported symptoms, mood, cognitive or physical functioning, and quality of life/participation within 12 months post-TBI. As the review aimed to explore the evidence on the recovery experience for the affected person, objective measures such as biomarkers and brain imaging were not included. Additionally, as the aim was to identify natural symptom experience and recovery, to prevent skewing the findings, intervention studies (designed to improve the subjective experience) were excluded.

Articles with both children and adults were included if data were split by age to enable extraction of adult (>16 years) data only. The same process was completed for severity of injury; articles which covered multiple injury severities were only included if mTBI data were able to be analyzed separately for outcome measures. Studies including biomarkers, imaging, or mTBI as an outcome, pharmacology, or rehabilitation were excluded. Studies exploring predictors or influences of outcomes were included if data from more than one time point were available. If multiple articles utilized the same data for analysis, only one was included to avoid duplication of results. Articles retrieved from the search were screened at the abstract level by one author (MB). Two reviewers then independently reviewed full-text articles against the inclusion criteria and resolved any difference in opinion through discussion. One author (MB) extracted the data. A third reviewer was available to resolve any disagreement in the selection process; however, this was not required. All eligible citations were extracted into EndNote (version X9.2). A data extraction form was created in Excel with column headings used to inform the data to be extracted. For the purposes of answering the research question, data were extracted on; first author, year of publication, country, study aim, if a pre-injury evaluation was conducted, details of the control group (if included), definition of mTBI, number of mTBI participants, age of the participants, mechanisms of injury included, gender, types of outcomes studied, outcome assessments used, timing of follow-ups, most common symptoms reported, as well as data on recovery time frames.

## Result

**Figure 1:**
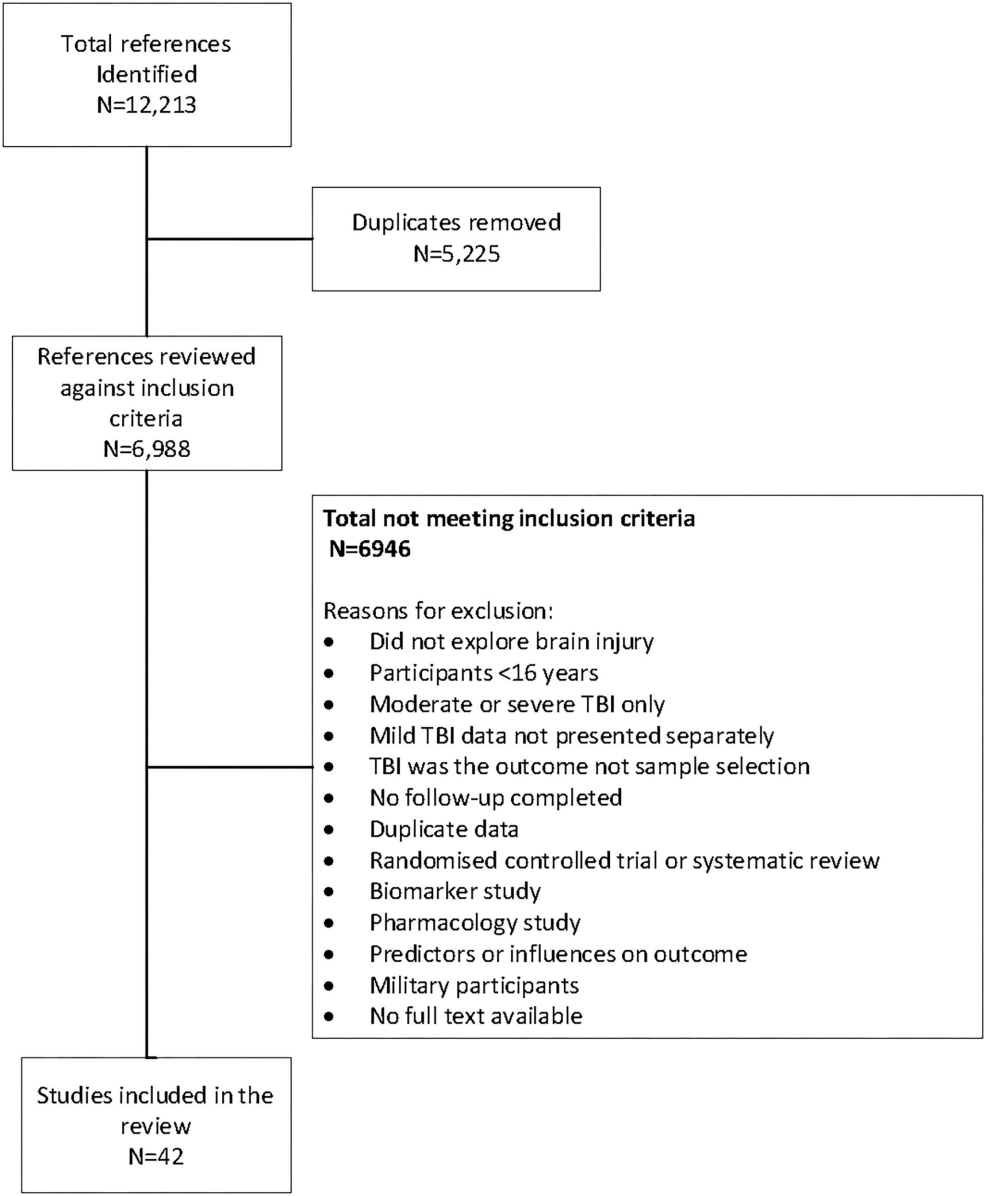
The study selection process.

A total of 12,212 references published up to March 10, 2021 were identified through the initial search of CINAHL (*n* = 1,329), Embase (*n* = 4,960), MEDLINE (*n* = 4,235), Pubmed (*n* = 1,002), and SPORTDiscus (*n* = 686) databases. After removal of duplicates, there were 6,974 abstracts for review. Of these, 174 full-text articles were reviewed. There were 42 articles that met the inclusion criteria. The selection process and reasons for exclusion are outlined in Figure 1.

On review of the included study populations, there were 18 studies that specifically presented data for sports-related injuries only (these were classified as sports-related studies and are shown in Table 2). There were 24 studies that provided data across all contexts and mechanisms of injury, including sports-related injuries (classified as general population studies and shown in Table 3). Only one study (36) directly presented data, comparing sports- vs. non-sports-related injuries using the same assessments at the same time points.

**Table 2:** Sports-related studies.

**First Author, Year; Country; Reference**	**Study sample characteristics (age range/ mean in years); gender; sport**	**Pre-injury Evaluation (Y/N); Control group included? (Y/N); Number of mTBI participants; Definition of mTBI**	**Outcomes studied**	**Tests used; Number of follow-ups: mean (range); Time of follow-up**	**Mean outcome scores; Common symptom presentation; Average recovery time**
Black et al., 2017; Canada (18)	Varsity athletes; (20); 44% male; women's rugby, men's football, women's hockey, men's basketball, men's hockey, field hockey.	Y; Y; 75; Concussion in Sport Group definition. Diagnosed by student therapist then sport physician.	Symptom recovery, cognitive recovery.	ImPACT, SCAT/ SCAT2; 11–12 (1–66); Daily (SCAT), once asymptomatic (ImPACT).	Outcome scores not included; Symptoms not included; 12.5 days (symptom recovery) 21.1 days (cognitive recovery).
Collins et al., 1999; USA (19)	Collegiate athletes; (20); 100% male; football.	Y; Y; 16; American Academy of Neurology grading. Diagnosed by athletic trainer then sport physician.	Neuropsychological test performance (verbal learning, delayed memory, visual scanning, executive functioning, attention, concentration, information processing speed, bilateral fine motor speed, word fluency), post-concussion symptoms.	HVLT, TMT A-B,Digit Span Test, SDMT, Grooved Pegboard Test, COWAT, PCSS; 4; within 24 h, 3, 5, 7 days PC.	HVLT: 24.6 (4.0) TMT-A: 21.0 (5.9) TMT-B: 55.4 (17.3) Digit Span: 15.8 (3.9) SDMT:56.8 (8.9) Grooved Pegboard dominant/nondominant: 67.1 (10.7)/ 73.5 (11.9) COWAT: 37.5 (9.3), PCSS: 10.3 (12.6); Symptoms not included; Recovery not included.
Covassin et al., 2007; USA (20)	Collegiate athletes; (college age); 52% male; wrestling, women's soccer, football, men's soccer, gymnastics, softball, lacrosse.	Y; N; 79; American Academy of Neurology grading. Diagnosed by athletic trainer then sport physician.	Cognitive functioning, post-concussion symptoms.	ImPACT; 2; Up to 3 days, 7–10 days.	Visual memory: 0.76, 0.74 Reaction time: 0.57, 0.53 Verbal memory: 0.85, 0.85 Processing speed: 38.81, 41.04 Total symptoms: 12.71, 12.53; Symptoms not included; Recovery not included.
Covassin et al., 2012; USA (21)	High school athletes; (19); mixed gender; football, women's soccer, men's soccer, women's volleyball, women's basketball, wrestling, men's basketball.	Y; N; 72; Concussion in Sport Group guidelines. Diagnosed by athletic trainer and sports physician.	Postural stability, post-concussion symptoms, cognitive performance.	ImPACT, BESS; 5; 2, 7, 14 days (ImPACT), 1, 2, 3 days (BESS).	BESS: 19.43 (1 day), 14.48 (3 days) Post concussion symptoms: 26.28 (2 days), 4.92 (14 days) Verbal memory processing: 80.89 (2 days), 82.26 (14 days) Visual memory: 67.18 (2 days), 68.15 (14 days) Reaction time: 0.648 (2 days), 0.578 (14 days) Motor processing speed: 36.52 (2 days), 40.74 (14 days); Symptoms not included; 7-14 days.
Echemendia et al., 2001; USA (22)	Collegiate athletes; (college age); mixed gender; football, men's soccer, men's ice hockey, women's basketball, men's basketball.	Y; Y; 29; Participants described as having experienced mTBI.	Post-concussion symptoms, memory, attention, visual and verbal functioning.	PCSC, HVLT, SDMT, Digit Span Test, Penn State Cancellation Test, TMT, COWAT, Stroop Test, Vigil Continuous Performance Test, five-word list-learning task; 4; 2, 48 h, 1 week, 1 month.	Symptom checklist: 8.53 (8.91) Digit span forward: 11.7 (1.46) Hopkins learning index: 0.71 (0.009) Hopkins delay index: 0.72 (0.14) Hopkins percent retained: 87.85 (13.32) List learning immediate recall index: 0.98 (0.02); Headache, nausea, dizziness, balance problems, drowsiness, sensitivity to light and noise, memory problems, problems concentrating; Recovery not included.
Fait et al., 2013; Canada (23)	Elite athletes; (20); 67% male; ice hockey, rugby, soccer.	N; Y; 6; Clinically diagnosed concussion by medical professional.	Locomotion, dual-task, cognitive function, information processing, attention, executive functioning, post-concussion symptoms.	Locomotion navigation task, Keatley Symptom Questionnaire, modified Stroop test, walking kinematic lab measures, Spatial and Digit Span Tests of Wechsler Memory Scale- 3rd edition, Brown-Peterson test, SDMT, TMT,	Maximum gait speed: 1.55m/s no obstacle, 1.51–1.53m/s with obstacle Stroop test with obstacle: 2–4 errors Minimum clearance: 0.3–0.58 Cognitive dual-task cost: no obstacle- 68.58 (42.98) with obstacle- 68.36–151.19 (55.87–121.18); Symptoms not included; Recovery not included.
				Color-word Interference Test of Delis-Kaplan Executive Function System, Test of Everyday Attention, California Computerized Assessment Package, Paced Auditory Serial Addition Test (neuropsychological tests); 2; 35–40 days (lab measures), neuropsychological test-w/in 14 days of lab measures.	
Field et al., 2003; USA (24)	High school and collegiate athletes; (20); 96% male; football, women's soccer.	Y; Y; 35; Defined by American Academy of Neurology Practice Parameter. Diagnosed by sports medicine practitioner.	Symptoms, verbal learning & memory, attention & concentration, speed of information processing, visual scanning & executive functioning, word fluency, visual memory (hs only).	PCSS, HVLT, Digit Span Test, SDMT, TMT A&B, COWAT; 4; within 24 h, 3, 5, 7 days.	Symptoms: 28.3 (24.5) HVLT total: 22.5 (4.3) HVLT delay: 6.5 (2.7); Symptoms not included; 7 days.
Guty et al., 2020; USA (25)	Collegiate athletes; (18–22); 50% male; soccer, football, lacrosse, basketball, wrestling, rugby.	Y; N; 40; diagnosed by university sports medicine staff.	Cognitive functioning, memory, executive functioning, attention, processing speed, post-concussion symptoms.	AWL: Total Immediate and Delay Recall, BVMT-R, SDMT, Digit Span Test, PSU Cancellation Test, Stroop Color-Word Test, BDI-FS, PCSS; 1; 6–36 months.	Memory composite: 98.66 (10.55) Attention/executive functioning composite: 99.09 (6.52); Headache, irritability, sadness, fatigue, sleep disturbance; Recovery not included.
McCrea et al., 2013; USA (26)	Athletes; (17); 89% male; football, soccer, lacrosse, ice hockey.	Y; Y; 570; American Academy of Neurology grading. Diagnosed by athletic trainer or sport physician.	Post-concussion symptoms, postural Stability, cognitive functioning.	GSC, BESS, SAC, HVLT, TMT Part B, SDMT, COWAT, Stroop test; 8; immediately, 3 h, 1, 2, 3, 5, 7, & 45 or 90 days.	Only reported as odds ratio; Symptoms not included; <7 days.
McCrea et al., 2003; USA (27)	Collegiate athletes; (20); 100% male; football.	Y; Y; 94; An injury resulting from a blow to the head causing an alteration in mental status, and at least one symptom from the American Academy of Neurology Guideline for Management of Sports Concussion. Diagnosed by sports physician or athletic trainer.	Post-concussion symptoms, cognitive impairment, postural stability, neurocognitive functioning.	GSC, SAC, BESS, HVLT, TMT-B, SDMT, COWAT, Stroop color-word test; 8; immediately, 3 h, 1, 2, 3, 5, 7, 90 days PC.	Only reported as odds ratio; Balance deficits; 7 days.
Meier et al., 2017; USA (28)	NCAA division 1 athletes; (20); 79% male; football, basketball, soccer, rowing, volleyball.	N; Y; 43; Diagnosed by sports medicine physician.	Behavior and cognitive function, mood, symptoms.	HAM-D, HAM-A, ANAM; 3; 1 day, 1 week, 1 month.	Only reported comparing timepoints; Symptoms not included; 13 days.
Nelson et al., 2016; USA (29)	High school and collegiate athletes; (16–20); 88% male; football, soccer, lacrosse, hockey.	Y; Y; 618; Defined by American Academy of Neurology Practice Parameter. Diagnosed by sports medicine practitioner.	Symptoms, cognitive functioning, postural stability.	GSC, SAC, BESS, HVLT, TMT-B, SDMT, Stroop test; 7; 3 h, 1, 2, 3, 5, 7, & 45 or 90 days.	Only includes estimated differences; Symptoms not included; 7 days.
Roiger et al., 2015 USA (30)	NCAA division 1 athletes; (20); 100% male; football, wrestling.	Y; Y; 7; Diagnosed by sports medicine physician.	Depression.	CES-D; 3; 1 week, 1 month, 3 months.	CES-D: 1 week 11.0, 1 month 8.3, 3 months 6.4; Depression; <1 month.
Turner et al., 2017; USA (31)	NCAA division 1 athletes; (19); 77% male; not described.	N; Y; 15; Diagnosed by sports medicine physician.	Mood, state anxiety.	POMS, STAI; 3; within 72 h, day 1 of exercise, date of return-to-play.	Tension: 2.21 (2.46) Anger: 1.43 (2.5) Fatigue: 5.14 (4.02) Depression: 1.64 (3.05) Vigor: 5.71 (6.02) Confusion: 3.29 (2.37) Total mood disturbance: 108.0 (15.48) State anxiety: 39.73 (12.84); Balance deficits; 7 days.
Vargas et al., 2015; USA (32)	Collegiate athletes; (18); 77% male; football, lacrosse, basketball, soccer, ice hockey, wrestling.	Y; Y; 84; Diagnosed by sports physician or athletic trainer.	Depression.	BDI-FS, PHIQ, WTAR, PCSS, ImPACT; 1; 48 h- 41 days (71% within 5 days).	Values reported as predictors of depression; Depression; Recovery not included.
Walton et al., 2021; USA (33)	Collegiate athletes; (19); 45% male; sport not described.	N; Y; 20; diagnosed by certified athletic trainer based on Concussion in Sport Group definition.	PCS, HRQOL, anxiety, fatigue, resilience, sleep disturbance.	HIS-r, TBI-QOL, Neuro-QOL; 3 or 4; within 72 h, 10 days, 17 days, after symptom free (if symptoms reported at third follow-up).	Anxiety: 14.5 Resilience: 36 Stigma: 9 Sleep disturbance: 17.5 Fatigue: 22 Appetite: 0; Anxiety, resilience, stigma, sleep disturbance; 6 days (4–10) (symptom recovery) 14 days (10–16) (full participation).
Wright et al., 2017; Canada (34)	Elite junior athletes; (19); 100% male; hockey, football.	Y; Y; 18; Diagnosed by sports physician, based on 4th International Conference on Concussion in Sport consensus statement.	Cerebral blood flow, symptom presence, symptom severity, balance, blood pressure.	SCAT3, SAC, BESS; 3; 3 days, 2 weeks, 1 month.	Number of symptoms: 11 (5.8) Symptom severity: 25.6 (20.6) SAC: 26.2 (2.3) BESS: 4.2 (3.1); Symptoms not included; 14 (7–35) days.
Zuckerman et al., 2012; US (35)	High school and collegiate athletes; (19); 39% male; football, soccer, basketball, softball.	Y; N; 100; On-field presentation of post-concussive symptoms. Diagnosed by athletic trainer or sports physician.	Return to baseline, symptoms, cognitive function.	ImPACT, PCSS; up to 2; 1–30 days.	Only includes baseline values; Symptoms not included; 5 days.

*ImPACT, Immediate Post-concussion Assessment and Cognitive Testing; SCAT, Sport Concussion Assessment Tool; HVLT, Hopkins Verbal Learning Test; TMT A&B, Trail-Making Tests A&B; SDM, Symbol Digit Modalities Test; COWAT, Controlled Oral Word Association Test; PCSS, Post-concussion Symptom Scale; BESS, Balance Error Scoring System; PCSC, Post-concussion Symptom Checklist; GSC, Graded Symptom Checklist; SAC, Standardized Assessment of Concussion; HAM-D & HAM-A, Hamilton Depression and Anxiety; ANAM, Automated Neuropsychological Assessment Metrics 4 Sports Medicine Battery; CES-D, Centre for Epidemiologic Studies Depression Scale; POMS, Profile of Mood States; STAI, State-Trait Anxiety Inventory; BDI-FS, Beck Depression Inventory- Fast Screen; PHIQ, Previous Head Injury Questionnaire; WTAR, Wechsler Test of Adult Reading*.

**Table 3:** Non-sports-related studies.

**First Author, Year; Country; Reference**	**Study sample characteristics: (age range/ mean in years); gender; injury setting**	**Pre-injury Evaluation (Y/N); Comparison group included? (Y/N); Number of mTBI participants; Definition of mTBI**	**Outcomes studied**	**Tests used; Number of follow-ups: mean (range); Time of follow-up**	**Mean outcome scores; Common symptom presentation; Average recovery time**
Beauchamp et al., 2020; Canada (36)	Adults presenting at emergency departments; (23–57); 61% male; skiing, snowboarding, hockey, soccer, football, motor vehicle collision, bicycle accident, pedestrian accident.	N; N; 1727; GCS 13–15.	Post-concussion symptoms.	RPCSQ; 3; 7, 30, 90 days.	Only reported as relative risks; Headache, confusion, poor concentration; Suggested within 90 days.
Chiang et al., 2016; Taiwan (37)	mTBI patients from a neurosurgical outpatient department; (20–81); 49% male; MVA, Pedestrian hit by vehicle, Fall, Hit by object.	N; N; 100; GCS 13–15.	Post-concussion symptoms, quality of life, outcome.	CPCS, GOSE, QOLIBRI, SF-36; 3; 1, 3, 12 months.	Not included; Dizziness, fatigue, headache, poor physical strength, poor memory, poor concentration; 54% by 1 year
Cicerone et al., 1995; USA (38)	Patients referred to a neuropsychology clinic due to persistent PCS; (18–61); 38% male; unknown injury setting.	N; N; 50; Alteration of mental status due to injury characterized by confusion, posttraumatic amnesia of <24 h, and loss of consciousness of <30 min.	Post-concussion symptoms, neuropsychological functioning, personality and emotional functioning, disability status after injury.	Post MTBI Symptom Checklist, MMPI, neuropsychological testing battery (attention, memory, language, reasoning, planning, organization); 1; 3–52 months (m-14 months).	Not included; Irritability, frustration, concentration, memory problems; 51% by 1 year.
Deb et al., 1998; UK (39)	Patients admitted to hospital after minor head injury; (18–93); 67% male; unknown injury setting.	N; N; 137; GCS 13–15.	Overall outcome, physical disability, cognitive state, premorbid IQ, psychiatric status, post-concussion symptoms.	GOS, ERSS, MMSE, NART, CIS-R, PSQ, behavior rating scale; 1; 1 year.	Not included; Symptoms not included; <1 year.
Emanuelson et al., 2003; Sweden (40)	Patients attending the Accident and Emergency Unit if the Department of General Surgery; (16–60); 65% male; Fall, hit by object, alcohol abuse, traffic.	N; Y; 101; <30 min loss of consciousness.	Quality of life, symptoms, health, well-being.	SF-36, PCSC; 3; 3 weeks, 3 months, 1 year.	At 1 year SF-36 scores- Physical functioning: 87.5 Role physical: 74.7 Bodily pain: 72.2 General health: 70.9 Vitality: 62.3 Social functioning: 83.2 Role emotional: 77.2 Mental health: 74.9 Physical composite score: 49.1 Mental composite score: 46.5 Number of symptoms at 1 year: 0–18 (mean 3.75); Tiredness, headache, neck pain, irritability, increased sleep, depression, anxiety; Recovery time not included
Fourtassi et al., 2011; Morocco (41)	Head trauma patients admitted to a teaching hospital; (18-64); 88% male; MVA, assault, fall, sport.	N; N; 42; GCS 13–15.	Post-concussion symptoms, quality of life.	PCL, VAS; 1; >1 year (m-15 months).	Severity of symptoms from PCL: difficulty remembering: 2.38 irritability: 2.28 fatigue: 2.28 noise sensitivity: 2.28 apathy: 1.97 headache: 1.88 argumentative: 1.69 depression: 1.54 anxiety: 1.43 doing things slowly: 1.40; Difficulty remembering, irritability, fatigue, noise sensitivity, depression, argumentative, apathy, headache, boredom, loneliness; >1 year
Hanks et al., 1999; USA (42)	Patients admitted to a Level 1 trauma centre; (21); 73% male; unknown injury setting.	N; Y; 138; GCS 13–15.	Emotional and behavioral changes, psychosocial adjustment.	KAS; 2; 1, 12 months.	KAS- anxiety: 8.28 belligerence: 5.92 sensory-perceptual distortions: 6.54 confusion: 3.97 helplessness: 6.48 hyperactivity: 4.69 negativity: 14.51 nervousness: 7.47 general psychopathology: 39.44 stability: 27.04 suspiciousness: 6.37 poor self-monitoring: 7.18 withdrawal: 10.53; Anxiety, belligerence, sensory-perceptual difficulties, confusion, helplessness, hyperactivity, negativism, nervousness, general adjustment difficulties; >1 year.
Heitger et al., 2007; New Zealand (43)	mTBI patients presenting to hospital; (15–56); 65% male; Sports, MVA, bicycle accident, fall.	N; Y; 37; GCS 13-15.	Symptoms.	RPQ, RHFUQ, SF-36v2; 4; 1 week, 3, 6, 12 months.	RPQ at 1 year- headache: 0.8 dizziness: 0.7 nausea: 0.2 noise sensitivity: 0.5 sleep disturbance: 0.7 fatigue: 0.9 irritability: 0.6 depression: 0.5 frustration: 0.6 poor memory: 0.8 poor concentration: 1.0 slowed thinking: 0.7 blurred vision: 0.7 light sensitivity: 0.5 double vision: 0.3 restlessness: 0.5; poor concentration, fatigue, taking longer to think, poor memory, headache; 3 months.
Hellstrøm et al., 2017; Norway (44)	mTBI patients admitted to university-affiliated trauma-referral centre; (16–65); 68% male; Traffic accident, fall, violence.	N; N; 62; GCS 13–15.	Post-concussion symptoms, global functioning, malingering, executive functioning.	RPQ, GOSE, FIT, WAIS-III, CWIT, FAS; 2; 4 weeks, 12 months.	RPQ total: 13.8 (15.2), 14.0 (13.1) GOSE: 7.0 (.856), 7.14 (.848); Symptoms not included; Recovery time not included.
Hsu et al., 2021; Taiwan (45)	Adults from outpatient clinics; (40–55); 39% male; traffic accident, ground-level fall, assault.	N; Y; 110; diagnosed by neurosurgeon, GCS 14–15.	Executive functioning, memory, information processing, depression, anxiety, irritability.	TWSLT, VFT, PASAT-R, BDI-II, BAI, NTUIS, CPCS; 2; 2 weeks, 3 years.	Long-term (mean: 2.9 years range: 6 months-6 years) follow-up CPCS- Physical: 4.21 Cognitive: 1.86 Emotional: 1.26 Total: 7.38; fatigue, loss of energy, insomnia, slowness of information processing, irritability, blurred vision; By long-term follow-up date.
Krpan et al., 2007; Canada (46)	TBI patients admitted to medical trauma centre; (34); 67% male; unknown injury setting.	N; Y; 8; GCS 13–15.	Coping strategies.	WOC-R; 1; 1 year.	WOC-R confrontive: 4.5 distancing: 6.0 self-controlling: 10.0 seeking social support: 5.1 accepting responsibility: 4.1 escape-avoidant: 6.6 planful problem-solving: 8.1 positive reappraisals: 5.8 total score: 8.1; Symptoms not included; Recovery time not included.
LecuyerGiguere et al., 2019; Canada (47)	mTBI patients presenting to Emergency Room; (18–55); 60% male; Sports, fall.	N; Y; 12; GCS 13-15.	Olfactory function, cognitive function, executive function, affective status, post-concussion symptoms.	Sniffin' Sticks Inventory Test, UPSIT, RBANS, DKEFS, TMT A-B, WAIS-IV, HADS, RPQ; 2; 24 h, 1 year.	UPSIT: 34.0, sniffin' sticks TDI at baseline: 31.1; hyposmia; >1year.
Lucas et al., 2016; USA (48)	mTBI patients enrolled at a Level 1 trauma centre within 1 week of injury; (44); 76% male; Vehicle, fall, violence, sports.	N; N; 212; GCS 13–15.	Prevalence of headache, depression, comorbid headache and depression.	PHQ-9; 2; 1 week, 1 year.	Not included; headache, depression; Suggested increase in symptoms at 1 year.
Losoi et al., 2016; Finland (49)	mTBI patients presenting at emergency department of hospital; (37); 61% male; Fall, sports, MVA, bicycle accident.	N; Y; 74; GCS 14–15.	Post-concussion symptoms, fatigue, insomnia, pain, post-traumatic stress, depression, quality of life, resilience, return-to-work.	GOSE, RPQ, BNI-FS, ISI, Pain Subscale of the RNBI, PTSD Checklist- Civilian version, BDI-II, RS, QOLIBRI, RAVLT, Stroop test, TMT A-B, Finger Tapping Test, WAIS-III, RTW; 3; 1, 6, 12 months.	At 1 year- RPCSQ: 6.9 BNI-FS: 8.1 ISI: 4.3 RNBI: 7.3 PCL-C: 23.3 RS: 143.3 QOLIBRI: 158.4 SWLS: 27; fatigue, insomnia, pain; 73.3% by 1 year.
McMahon et al., 2014; USA (50)	Patients at one of three Level 1 trauma centres;(18–94); 70% male; unknown injury setting.	N; N; 375; GCS 13–15.	Post-concussion symptoms, post-TBI outcome.	GOSE, BSI-18, RPQ, SWLS,PCSC; 3; 3, 6, 12 months.	At 1 year- total PCS symptoms: 6.8 physical symptoms: 2.8 cognitive symptoms: 1.7 emotional symptoms: 1.3 sleep symptoms: 1.1; Symptoms not included; Suggested increase in some symptoms at 1 year
Nelson et al., 2019; USA (12)	Patients presenting to level 1 trauma centres; (Mean age 41); (66% male); Vehicle accidents, fall, assault and other.	N; Y; 1154; GCS 13–15.	Functioning, post-concussion symptoms, psychological distress.	GOSE, RPQ, BSI; 4; 2 weeks, 3, 6, 12 months.	Only reported as percentage prevalence; headache, fatigue, depression, forgetfulness; 47.2% by 1 year.
Oldenburg et al., 2018; Sweden (51)	Patients at any of three emergency departments; (15–65); 61% male; Fall, traffic, assault.	N; N; 94; GCS 13–15.	Post-concussion symptoms, neurological disorders, stress reaction, preinjury behavior and personality.	RPQ, RHFUQ, Axis I-V of DSM-IV, HADS, IES-R, SSP, SOC, AUDIT; 1; 1 week, 1 year.	At 1 year for recovered group- IES-R: intrusions: 6.2 avoidance: 4.8 hyperarousal: 3.5 total: 14.5 HADS: anxiety: 2.7 depression: 2.2 for group reporting symptoms at 1 year- IES-R: intrusions: 15.2 avoidance: 9.3 hyperarousal: 12.4 total: 36.8 HADS: anxiety: 8.4 depression: 6.7; Symptoms not included; 88% symptom-free at 1 year.
Røe et al., 2009; Norway (52)	Patients enrolled at the neurosurgical department of a hospital; (16–60); 62% male; Traffic, fall, violence.	N; N; 96; GCS 13–15.	Post-concussion symptoms.	RPQ; 4; Within 48 h, 3, 6, 12 months.	Not included; Headache, dizziness, fatigue, noise sensitivity, sleep disturbance, forgetfulness, poor concentration; Suggested increase in some symptoms at 1 year.
Sigurdardottir et al., 2009; Norway (53)	Patients at a Level 1 trauma centre; (16–55); 62% male; Traffic, fall, assault.	N; Y; 40; GCS 13–15.	Post-concussion symptoms, neuropsychological functioning.	RPQ, HADS, GOAT; 3; Within 24 h, 3, 12 months.	At 3 months- RPQ: 20.8 (18.3) HADS: 6.9 (4.9) At 1 year- RPQ: 15.9 (16.9) HADS: 5.4 (4.6); Headache, fatigue, frustration, memory problems, concentration problems, taking longer to think, restlessness; 72.7% by 1 year.
Skilbeck et al., 2013; Australia (54)	Patients included in the Tasmanian Neurotrauma Register; (16–83); 61% male; Transport-related; fall, assault, sport.	N; N; 172; GCS 13–15.	Full Scale IQ, anxiety, depression.	NART, HADS; 3; 1, 6, 12 months.	Estimated FSIQ at 1 year- 106.28 (8.47); Symptoms not included; <6 months.
Steward et al., 2016; USA (55)	Patients from University of Alabama School of Medicine; (19–79); 55% male; MVA, fall, other vehicle accident.	N; Y; 51; GCS 13–15.	Medical decision-making capacity, capacity to consent, reasoning, understanding	CCTI, GOAT; 3; 1, 6, 12 months	GOAT at 1 year- mTBI: 93.08 complicated mTBI: 97.38. CCTI at 1 year- mTBI [complicated mTBI]: expressing choice: 4.0 [3.94] reasonable choice: 0.96 [1.0]
					appreciation: 7.08 [7.13] reasoning: 8.81 [8.13] understanding: 61.23 [60.94]; Poor appreciation, reasoning, and understanding; 6-12 months
Theadom et al., 2016; New Zealand (13)	mTBI patients residing in Hamilton or Waikato districts; (38); 59% male; Fall, MVA, exposure to mechanical force, assault.	N; N; 342; GCS 13–15.	Post-concussion symptoms, quality of life, cognitive functioning, depression, anxiety, overall functioning.	RPQ, GOS, CNS-VS, HADS, SF-36; 4; within 2 weeks, 1, 6, 12 months.	Reported as percentage/number of participants; headache, fatigue, forgetfulness, poor concentration, taking longer to think; >50% by 1 year.
Singh et al., 2019; UK (56)	TBI patients presenting to emergency department of teaching hospital; (17–94); 69% male; Fall, traffic, assault.	N; N; 651; GCS 12–15	Post-concussion symptoms, return-to-work, functioning, global outcome.	GOSE, RHFUQ, HADS, RPQ, RTW; 2; 10 weeks, 1 year.	GOSE at 1 year (number of participants): dead: 20 severe lower: 1 severe upper: 38 moderate lower: 88 moderate upper: 109 good lower: 129 good upper: 215. Return-to-work at 1 year- full: 345 partial: 149 none: 86; Symptoms not included; Recovery not included.
Sterr et al., 2006; UK (57)	mTBI patients attending one of 150 general practitioner surgeries, a local Brain Injury Community Centre, or a University campus; (18–65); 63% male; Fall, sport, traffic, assault.	N; Y; 38; Loss of consciousness <30 min, post traumatic amnesia <24 h, alteration of mental state (dazed, disorientated, confused) at time of incident.	Post-concussion symptoms, cognitive functioning, attention, IQ.	RPQ, CFQ, TAP, CANTAB, NART; 1; 12+ months.	Only included in graphic form; Headache, noise sensitivity, sleep disturbance, fatigue, irritability, depression, frustration, memory issues, poor concentration, taking longer to think; 71% within 1 year.

*CPSC, Checklist of post-concussion syndrome; GOSE, Glasgow Outcome Scale- Extended; QOLIBRI, Quality of life after brain injury; SF-36, Short Form 36 Heath Survey; MMPI, Minnesota Multiphasic Personality Inventory; GOS, Glasgow outcome scale; ERSS, Edinburgh rehabilitation status scale; MMSE, mini mental state examination; NART, National adult reading test; CIS-R, clinical interview schedule-revised; PSQ, psychosis screening questionnaire; PCSC, post-concussion symptom checklist; PCL, Problem checklist; VAS, visual analogue scale; RPQ, Rivermead Post-concussion Symptoms Questionnaire; FIT, Rey Fifteen-Item Test; WAIS-III, Wechsler Adult Intelligence Scale Third Edition; CWIT, Color Word Interference Test; FAS, Letter Fluency Task; WOC-R, Ways of Coping Questionnaire- Revised; UPSIT, University of Pennsylvania Smell Identification Test; RBANS, Repeatable Battery for the Assessment of Neuropsychological Status; DKEFS, Delis-Kaplan Executive Function System; TMT- A&B, Trail Making Test A& B; HADS, Hospital Anxiety and Depression Scale; PHQ-9, Patient Health Questionnaire-9; BSI-18, Brief Symptom Inventory-18; SWLS, Satisfaction with Life Scale; RHFUQ, Rivermead Head Injury Follow-up Questionnaire; IES-R, Impact of Event Scale- Revised; SSP, Swedish Universities Scales of Personality; SOC, Sense of Coherence Scale; AUDIT, Alcohol Use Disorders Identification Test; GOAT, Galveston Orientation and Amnesia Test; CCTI, Capacity to Consent to Treatment Instrument; CNS-VS, CNS Vital Signs; BNI-FS, Barrow Neurological Institute Fatigue Scale; ISI, Insomnia Severity Index; RNBI, Ruff Neurobehavioral Inventory; BDI-II, Beck Depression Inventory- Second Edition; RS, Resilience Scale; RAVLT, Rey Auditory Verbal Learning Test; RTW, Return to Work; KAS, Katz Adjustment Scale; CFQ, Cognitive Failures Questionnaire; TAP, Test of Attentional Performance; CANTAB, Cambridge Neuropsychological Test Automated Battery*.

The 42 studies meeting inclusion criteria underwent data extraction using a standardized form for information about participant demographics, number and timing of follow-ups sessions, testing methods, and outcomes measured. For the sports-related articles, information on the type of sport was included, while the mechanism of injury was included for non-sport articles (Tables 2, 3).

Description of the severity of injury was determined based on the reported Glasgow Coma Scale (GCS) score (58) in the non-sport injury-related articles, with a score of 13–15 being defined as a mild TBI. Three studies (38, 40, 57) did not include the GCS score and described severity based on duration of post-traumatic amnesia (<24 h) and loss of consciousness (<30 min) immediately post-injury. Sport-related articles did not report the GCS score. Instead, severity was based on description of the injury by an athletic trainer or a sports physician. Nine studies also graded individuals based on the American Academy of Neurology or Concussion in Sport Group guidelines (18–21, 24, 26, 27, 29, 34).

Of 18 sports-related mTBI studies included, only one (23) did not have participants who played football, and two (31, 33) did not include information about the type of sports played. Fifteen studies originated from the USA with the other three (18, 23, 34) from Canada. The number of mTBI participants per study ranged from 6 to 570, with a median of 58 participants. The proportion of male participants in sport studies varied from 37 to 100%, with more than 50% male participant groups in 13 of 18 studies. Four studies (23, 28, 31, 33) did not include pre-injury evaluations, and different four (20, 21, 25, 35) did not include a control or comparison group.

From the 24 general population mTBI articles, the mechanism of injury was most commonly from a fall or a motor vehicle accident. Five studies (38, 39, 42, 46, 50) did not state the mechanism or setting of injury but did not relate incidents to sporting environments. Eleven studies (40, 42, 43, 45–47, 49, 53–55, 57) included a control or a comparison group of differing injury severities. Almost half of included studies originated from the USA, UK, or Norway (*n* = 12) (12, 38, 39, 42, 44, 48, 50, 52, 53, 55–57) with the remainder originating from various countries across North America, Europe, Oceania, and Asia. The number of participants included in each study that participated in each follow-up session ranged from 8 to 1,727 (median = 94). Only three studies (37, 38, 45) had <50% male participant groups.

The most common outcomes measured for sports mTBI were post-concussion symptoms, depression, and cognitive functioning, while the most common outcomes assessed in non-sports mTBI studies were post-concussion symptoms, quality of life, cognitive and executive functioning, and personality or behavioral changes. To measure these outcomes, sports-related studies frequently used the Graded Symptom Checklist (GSC), Post-concussion Symptom Scale (PCSS), and the Immediate Post-Concussion Assessment and Testing (ImPACT), while general population studies used the Rivermead Post-concussion Symptoms Questionnaire (RPQ), Glasgow Outcome Scale [Extended] (GOS/GOSE), Hospital Anxiety and Depression Scale (HADS), and the 36-Item Short Form Survey (SF-36).

The mean number of follow-up sessions in general population studies was two, while the sport studies averaged four follow-up time points. Only six general population studies (43, 45, 47, 48, 51–53) conducted the first follow-up appointment within 1 week of injury, while fifteen of the sports mTBI studies conducted their first follow-up within this time frame. Time of follow-ups ranged from <3 h to 36 months post-mTBI in the sports studies, with 11 studies (22, 23, 25–30, 32, 34, 35) including a follow-up testing session at least 1-month post-injury or later. Of those 11, only six studies (23, 25–27, 29, 30) included the latest follow-up time point of 90 days or 3 months. All but 1 of the 24 general population studies (36) included a follow-up at 12 months or later, with the earliest follow-up within 24 h post-injury. The most frequent follow-up times <12 months were at 1 month (*n* = 8) and 6 months (*n* = 8).

In the sports studies, the mean age of participants ranged from 17 to 20, while the general population studies mean age was older at 30–40 years. Of the five non-sport studies (36, 43, 47, 49, 57) that included sports as a mechanism of injury in at least 10% of participants, only one study (36) directly compared outcomes of sports vs. non-sports-related mTBI participants. No comparison was made by other authors between groups of sports vs. non-sport, and the participants were not differentiated or split in data in a way to enable these comparisons to be made. The sole study comparing sports vs. non-sports (36) investigated prevalence of post-concussion symptoms and return to daily activities at 7- and 90-day post-mTBI based on results from the Rivermead Post-concussion Questionnaire. This study revealed no differences in overall symptom burden, mood, or recovery at 7- and 90-days post-injury (36).

Due to the lack of comparable data due to use of different outcome measures and follow-up time frames, we were not able to compare disease burden or recovery time frames between the two contexts apart from using data obtained within one study (36).

## Discussion

This scoping review highlighted some key methodological differences in how studies are conducted between sports and non-sports related mTBI, making comparisons in symptom presentation and impact between the two contexts difficult. The review has highlighted a significant gap in the current literature, given the key differences in current evidence for the definitions used, outcome measures used to assess impact such as symptoms and cognitive function, and timing of outcome assessments. There is a need for collaboration and consensus between the two fields to enable greater understanding of the similarities and differences of sports- and non-sports-related injuries to guide clinical decision-making.

One key difference between the two contexts was the way injuries were described and classified. In the non-sports context, mTBIs were assessed and framed in terms of injury severity by GCS score, whereas, in contrast, there was no reference to the broader context or how it was decided this was a “concussive” injury and not of moderate severity within the sports context. Consequently, it was not clear which types of injuries were excluded from mild SR-TBI studies. Given moderate to severe injuries can be sustained within sports context (59), this may be something that sports physicians and academics consider, record, and report on. In updated versions of commonly used SR-TBI assessments, such as the SCAT-5, one portion of the assessment tool includes conducting the GCS (60). Given ambulance personnel routinely use GCS in most countries, this could easily be reported on SR-TBI studies to assist in study comparison.

One consideration in conducting this review was that, often, general population studies included SR-TBI within a larger dataset. However, these injuries were often classified under different mechanism categories (e.g., falls and being hit by an object), whereas SR-TBI studies focused on injuries specifically on any injury sustained during sport. It was not possible to combine data from these studies to explore differences in symptom presentation and recovery time frames due to the methodological and reporting differences. For example, general population studies presented data that between 47 and 88% of participants had recovered by 1 year. In contrast, the sports-related studies reported findings that average days to recovery were between 7 and 14 days. However considerable caution needs to be taken to directly interpret these findings as the way recovery was defined and measured was not comparable and restricted by few longer-term follow-ups in sports-related mTBI studies. The only direct comparison (36) made between sports- and non-sports-related mTBI groups found few differences in symptom presentation between groups. Whilst there were no differences in overall symptom burden or recovery between the two groups in this study, on a symptom-specific level, SR-TBI participants were at a greater risk of impaired concentration compared to non-sports participants in the week following mTBI, and exhibited fewer and less severe symptoms such as fatigue and dizziness at the later follow-up. This suggested that there may be small discrepancies between sports and non-sports mTBI presentation for some symptoms. However, additional studies are needed to compare symptom presentation and recovery time frames between the two contexts to determine if these injuries do need to be considered separately or whether a blended model, with embedded flexibility, may be a more effective and simpler health care pathway.

Additionally, discrepancy between studies within the two contexts was the lack of long-term follow-ups in sports-related mTBI studies compared to studies on the general population. Previous epidemiology research (13) showed significant prevalence of post-concussion symptoms up to 12 months post-mTBI in a community population including sports-related injuries. However, this review found that individuals who had suffered an SR-TBI were very rarely assessed for any mTBI-related outcomes beyond 3 months post-injury. Whilst many patients do recover relatively quickly following an mTBI (5), there is likely to be a proportion who experiences an SR-TBI who may still experience difficulties beyond 3 months, particularly if there was significant mTBI history. There is evidence that those who have experienced prior injuries have been more at risk of a prolonged recovery (61). Case studies were presented where sports athletes were report long-term and cumulative impacts (62). Increasing follow-up length in SR-TBI-related studies would help identify risk factors in longer-term problems and key intervention points in recovery trajectories where more intensive support is required to facilitate recovery. Although not conclusive, the findings from this review highlighted that information about possible long-term effects of an mTBI was lacking within the sports context.

There was a considerable overlap in the types of outcome domains studies, e.g., symptom presentation, cognitive function, and mood; however, the diversity in outcome measures used to assess these domains prevented direct comparison of the findings. Whilst there is a clear benefit in researching specific subpopulations in detail; there is also a need to look more broadly across the disorder spectrum. This review highlights a need for integration and collaboration across these two fields of enquiry to advance the field. The neurophysiological trauma to the brain is likely similar no matter whether the mechanisms are from sports-related or non-sports-related activities. Therefore, further evidence is needed to determine if different approaches to assessment and management should be similar or different across injury contexts.

### Limitations

The key limitation of this review related to the search terms included. Overly broad terms such as “outcome” or “recovery” were not included to decrease the high number of irrelevant articles that were identified when including these terms. However, this may have inadvertently resulted in the exclusion of relevant studies. Since completion of the review, other relevant studies have been published but were not able to be considered within this review such as the Concussion Assessment, Research, and Education Consortium research (63). However, the key differences in methods used between these two fields of study are important to highlight to guide future research and to ensure optimal health care pathways are developed to support patients most effectively. Additionally, the sample size for some studies was low. Once the gap in the current literature has been addressed, it will be important to conduct a systematic review including of study quality to identify if there are similarities and differences in self-reported recovery following sports and non-sports-related injuries.

## Conclusion

This review highlighted a key gap in the current literature, comparing recovery trajectories across different contexts of injury and has reflected a need for greater collaboration between sports and general population fields of research. Future research should focus on longer-term follow-ups, particularly in sports-related studies, and utilize a more standardized method of assessing mTBI- affected individuals to inform optimal treatment approaches.

## Data Availability Statement

The original contributions presented in the study are included in the article/supplementary material, further inquiries can be directed to the corresponding author.

## Author Contributions

All authors listed have made a substantial, direct, and intellectual contribution to the work and approved it for publication.

## Funding

This review was conducted with support from an AUT, Faculty of Health and Environmental Sciences Summer Scholarship Grant. The funder was not involved in the study design or interpretation of the findings. Funds for open access publication fees were provided by the AUT TBI Network.

## Conflict of Interest

The authors declare that the research was conducted in the absence of any commercial or financial relationships that could be construed as a potential conflict of interest.

## Publisher's Note

All claims expressed in this article are solely those of the authors and do not necessarily represent those of their affiliated organizations, or those of the publisher, the editors and the reviewers. Any product that may be evaluated in this article, or claim that may be made by its manufacturer, is not guaranteed or endorsed by the publisher.

## References

[1] Menon DK Schwab K Wright DW Maas AI. Position statement: definition of traumatic brain injury. Arch Phys Med Rehabil. (2010) 91:1637–40. 10.1016/j.apmr.2010.05.017 21044706

[2] Harmon KG Drezner J Gammons M Guskiewicz K Halstead M Herring S . American medical society for sports medicine position statement: concussion in sport. Clin J Sport Med. (2013) 23:1–18. 10.1097/JSM.0b013e31827f5f93 23269325

[3] West TA Marion DW. Current recommendations for the diagnosis and treatment of concussion in sport: a comparison of three new guidelines. J Neurotrauma. (2014) 31:159–68. 10.1089/neu.2013.3031 23879529 PMC3900013

[4] Sharp DJ Jenkins PO. Concussion is confusing us all. Pract Neurol. (2015) 15:172–86. 10.1136/practneurol-2015-001087 25977270 PMC4453625

[5] McCrory P Meeuwisse W Dvorák J Aubry M Bailes J Broglio S . Consensus statement on concussion in sport—the 5th international conference on concussion in sport held in Berlin, October 2016. Br J Sports Med. (2017) 51:838–47. 10.1136/bjsports-2017-097699 28446457

[6] Hume PA King D McGeown J Theadom A. Sports-related concussion, mild traumatic brain injury or sport-originated brain injury (SOBI): a more useful term. N Z J Sports Med. (2019) 45:64–7.

[7] Maas AIR Menon DK Adelson PD Andelic N Bell MJ Belli A . Traumatic brain injury: integrated approaches to improve prevention, clinical care, and research. Lancet Neurol. (2017) 16:987–1048. 10.1016/S1474-4422(17)30371-X 29122524

[8] Dewan MC Rattani A Gupta S Baticulon RE Hung YC Punchak M . Estimating the global incidence of traumatic brain injury. J Neurosurg. (2018) 130:1–1097. 10.3171/2017.10.JNS17352 29701556

[9] Feigin VLP Theadom AP Barker-Collo SP Starkey NJP McPherson KP Kahan MMB . Incidence of traumatic brain injury in New Zealand: a population-based study. Lancet Neurol. (2013) 12:53–64. 10.1016/S1474-4422(12)70262-4 23177532

[10] Cnossen M Polinder S Allanson J Anke A Audibert G Badenes R . Variation in structure and process of care in traumatic brain injury: provider profiles of European neurotrauma centers participating in the CENTER-TBI study. PLoS ONE. (2016) 11:e0161367. 10.1371/journal.pone.0161367 27571205 PMC5003388

[11] Marshall S Bayley M McCullagh S Berrigan L Fischer L Ouchterlony D . Guideline for Concussion/Mild Traumatic Brain Injury and Persistent Symptom (for Adults 18+ years of age). 3rd ed Toronto, ON: Ontario Neurotrauma Foundation (2018).

[12] Nelson LD Temkin NR Dikmen S Barber J Giacino JT Yuh E . Recovery after mild traumatic brain injury in patients presenting to US level i trauma centers: a transforming research and clinical knowledge in traumatic brain injury (TRACK-TBI) study. JAMA Neurol. (2019) 76:1049–59. 10.1001/jamaneurol.2019.1313 31157856 PMC6547159

[13] Theadom A Parag V Dowell T McPherson K Starkey N Barker-Collo S . Persistent problems 1 year after mild traumatic brain injury: a longitudinal population study in New Zealand. Br J Gen Pract. (2016) 66:e16–23. 10.3399/bjgp16X683161 26719482 PMC4684031

[14] Möller MC Lexell J Wilbe Ramsay K. Effectiveness of specialized rehabilitation after mild traumatic brain injury: a systematic review and meta-analysis. J Rehabil Med. (2021) 53:jrm00149. 10.2340/16501977-2791 33492404 PMC8814853

[15] Klein AP Tetzlaff JE Bonis JM Nelson LD Mayer AR Huber DL . Prevalence of potentially clinically significant magnetic resonance imaging findings in athletes with and without sport-related concussion. J Neurotrauma. (2019) 36:1776–85. 10.1089/neu.2018.6055 30618331 PMC6551984

[16] Manley G Gardner AJ Schneider KJ Guskiewicz KM Bailes J Cantu RC . A systematic review of potential long-term effects of sport-related concussion. Br J Sports Med. (2017) 51:969–77. 10.1136/bjsports-2017-097791 28455362 PMC5466926

[17] Munn Z Peters MDJ Stern C Tufanaru C McArthur A Aromataris E. Systematic review or scoping review? guidance for authors when choosing between a systematic or scoping review approach. BMC Med Res Methodol. (2018) 18:143. 10.1186/s12874-018-0611-x 30453902 PMC6245623

[18] Black AM Sergio LE Macpherson AK. The epidemiology of concussions: number and nature of concussions and time to recovery among female and male canadian varsity athletes 2008 to 2011. Clin J Sport Med. (2017) 27:52–6. 10.1097/JSM.0000000000000308 26862834

[19] Collins MW Grindel SH Lovell MR Dede DE Moser DJ Phalin BR . Relationship between concussion and neuropsychological performance in college football players. JAMA. (1999) 282:964–70. 10.1001/jama.282.10.964 10485682

[20] Covassin T Schatz P Swanik CB. Sex differences in neuropsychological function and post-concussion symptoms of concussed collegiate athletes. Neurosurgery. (2007) 61:345–50. 10.1227/01.NEU.0000279972.95060.CB 17762747

[21] Covassin T Elbin RJ Harris W Parker T Kontos A. The role of age and sex in symptoms, neurocognitive performance, and postural stability in athletes after concussion. Am J Sports Med. (2012) 40:1303–12. 10.1177/0363546512444554 22539534

[22] Echemendia RJ Putukian M Mackin RS Julian L Shoss N. Neuropsychological test performance prior to and following sports-related mild traumatic brain injury. Clin J Sport Med. (2001) 11:23–31. 10.1097/00042752-200101000-00005 11176142

[23] Fait P Swaine B Cantin JF Leblond J McFadyen BJ. Altered integrated locomotor and cognitive function in elite athletes 30 days postconcussion: a preliminary study. J Head Trauma Rehabil. (2013) 28:293–301. 10.1097/HTR.0b013e3182407ace 22495102

[24] Field M Collins MW Lovell MR Maroon J. Does age play a role in recovery from sports-related concussion? a comparison of high school and collegiate athletes. J Pediatr. (2003) 142:546–53. 10.1067/mpd.2003.190 12756388

[25] Guty E Riegler K Meyer J Walter AE Slobounov SM Arnett P. Symptom factors and neuropsychological performance in collegiate athletes with chronic concussion symptoms. Arch Clin Neuropsychol. (2020) 36: 746–56. 10.1093/arclin/acaa092 33140096

[26] McCrea M Guskiewicz K Randolph C Barr WB Hammeke TA Marshall SW . Incidence, clinical course, and predictors of prolonged recovery time following sport-related concussion in high school and college athletes. J Int Neuropsychol Soc. (2013) 19:22–33. 10.1017/S1355617712000872 23058235

[27] McCrea M Guskiewicz KM Marshall SW Barr W Randolph C Cantu RC . Acute effects and recovery time following concussion in collegiate football players: the NCAA concussion study. JAMA. (2003) 290:2556–63. 10.1001/jama.290.19.2556 14625332

[28] Meier TB Bellgowan PSF Mayer AR. Longitudinal assessment of local and global functional connectivity following sports-related concussion. Brain Imaging Behav. (2017) 11:129–40. 10.1007/s11682-016-9520-y 26821253

[29] Nelson LD Guskiewicz KM Barr WB Hammeke TA Randolph C Ahn KW . Age differences in recovery after sport-related concussion: a comparison of high school and collegiate athletes. J Athl Train. (2016) 51:142–52. 10.4085/1062-6050-51.4.04 26974186 PMC4852320

[30] Roiger T Weidauer L Kern B. A longitudinal pilot study of depressive symptoms in concussed and injured/nonconcussed national collegiate athletic association division i student-athletes. J Athl Train. (2015) 50:256–61. 10.4085/1062-6050-49.3.83 25562455 PMC4477920

[31] Turner S Langdon J Shaver G Graham V Naugle K Buckley T. Comparison of psychological response between concussion and musculoskeletal injury in collegiate athletes. Sport Exerc Perform Psychol. (2017) 6:277–88. 10.1037/spy0000099 29250458 PMC5726592

[32] Vargas G Rabinowitz A Meyer J Arnett PA. Predictors and prevalence of postconcussion depression symptoms in collegiate athletes. J Athl Train. (2015) 50:250–5. 10.4085/1062-6050-50.3.02 25643158 PMC4477919

[33] Walton SR Broshek DK Kranz S Malin SK Hertel J Resch JE. Mood, psychological, and behavioral factors of health-related quality of life throughout recovery from sport concussion. J Head Trauma Rehabil. (2021) 36:128–36. 10.1097/HTR.0000000000000604 32769824

[34] Wright AD Smirl JD Bryk K van Donkelaar P. A prospective transcranial doppler ultrasound-based evaluation of the acute and cumulative effects of sport-related concussion on neurovascular coupling response dynamics. J Neurotrauma. (2017) 34:3097–106. 10.1089/neu.2017.5020 28627298

[35] Zuckerman SL Lee YM Odom MJ Solomon GS Forbes JA Sills AK. Recovery from sports-related concussion: days to return to neurocognitive baseline in adolescents versus young adults. Surg Neurol Int. (2012) 3:130. 10.4103/2152-7806.102945 23227435 PMC3513851

[36] Beauchamp F Boucher V Neveu X Ouellet V Archambault P Berthelot S . Post-concussion symptoms in sports-related mild traumatic brain injury compared to non-sports-related mild traumatic brain injury. CJEM. (2021) 23:223–31. 10.1007/s43678-020-00060-0 33512694

[37] Chiang CC Guo SE Huang KC Lee BO Fan JY. Trajectories and associated factors of quality of life, global outcome, and post-concussion symptoms in the first year following mild traumatic brain injury. Qual Life Res. (2016) 25:2009–19. 10.1007/s11136-015-1215-0 26706751

[38] Cicerone KD Kalmar K. Persistent postconcussion syndrome: the structure of subjective complaints after mild traumatic brain injury. J Head Trauma Rehabil. (1995) 10:1–17. 10.1097/00001199-199510030-00002

[39] Deb S Lyons I Koutzoukis C. Neuropsychiatric sequelae one year after a minor head injury. J Neurol Neurosurg Psychiatry. (1998) 65:899–902. 10.1136/jnnp.65.6.899 9854967 PMC2170392

[40] Emanuelson I Andersson Holmkvist E Björklund R Stålhammar D. Quality of life and post-concussion symptoms in adults after mild traumatic brain injury: a population-based study in western Sweden. Acta Neurol Scand. (2003) 108:332–8. 10.1034/j.1600-0404.2003.00155.x 14616303

[41] Fourtassi M Hajjioui A Ouahabi AE Benmassaoud H Hajjaj-Hassouni N Khamlichi AE. Long term outcome following mild traumatic brain injury in Moroccan patients. Clin Neurol Neurosurg. (2011) 113:716–20. 10.1016/j.clineuro.2011.07.010 21840643

[42] Hanks RA Temkin N Machamer J Dikmen SS. Emotional and behavioral adjustment after traumatic brain injury. Arch Phys Med Rehabil. (1999) 80:991–7. 10.1016/S0003-9993(99)90049-7 10488997

[43] Heitger MH Jones RD Frampton CM Ardagh MW Anderson TJ. Recovery in the first year after mild head injury: divergence of symptom status and self-perceived quality of life. Journal of Rehabil Med. (2007) 39:612–21. 10.2340/16501977-0100 17896052

[44] Hellstrøm T Westlye LT Sigurdardottir S Brunborg C Soberg HL Holthe Ø . Longitudinal changes in brain morphology from 4 weeks to 12 months after mild traumatic brain injury: associations with cognitive functions and clinical variables. Brain Injury. (2017) 31:674–85. 10.1080/02699052.2017.1283537 28414250

[45] Hsu HH Lai WH Yu HT Xiao SH Tsai YH Wang KC . Long-term presentation of postconcussion symptoms and associated factors: analysis of latent class modeling. Arch Clin Neuropsychol. (2021) 36:62–73. 10.1093/arclin/acaa063 32839820

[46] Krpan KM Levine B Stuss DT Dawson DR. Executive function and coping at one-year post traumatic brain injury. J Clin Exp Neuropsychol. (2007) 29:36–46. 10.1080/13803390500376816 17162720

[47] Lecuyer Giguère F Frasnelli A De Guise É Frasnelli J. Olfactory, cognitive and affective dysfunction assessed 24 hours and one year after a mild Traumatic Brain Injury (mTBI). Brain Injury. (2019) 33:1184–93. 10.1080/02699052.2019.1631486 31223039

[48] Lucas S Smith BM Temkin N Bell KR Dikmen S Hoffman JM. Comorbidity of headache and depression after mild traumatic brain injury. Headache. (2016) 56:323–30. 10.1111/head.12762 26814846

[49] Losoi H Silverberg ND Wäljas M Turunen S Rosti-Otajärvi E Helminen M . Recovery from mild traumatic brain injury in previously healthy adults. J Neurotrauma. (2016) 33:766–76. 10.1089/neu.2015.4070 26437675

[50] McMahon P Hricik A Yue JK Puccio AM Inoue T Lingsma HF . Symptomatology and functional outcome in mild traumatic brain injury: results from the prospective TRACK-TBI study. J Neurotrauma. (2014) 31:26–33. 10.1089/neu.2013.2984 23952719 PMC3880097

[51] Oldenburg C Lundin A Edman G Deboussard CN Bartfai A. Emotional reserve and prolonged post-concussive symptoms and disability: a Swedish prospective 1-year mild traumatic brain injury cohort study. BMJ Open. (2018) 8:e020884. 10.1136/bmjopen-2017-020884 29982209 PMC6042551

[52] Røe C Sveen U Alvsåker K Bautz-Holter E. Post-concussion symptoms after mild traumatic brain injury: influence of demographic factors and injury severity in a 1-year cohort study. Disabil Rehabil. (2009) 31:1235–43. 10.1080/09638280802532720 19116810

[53] Sigurdardottir S Andelic N Roe C Jerstad T Schanke A. Post-concussion symptoms after traumatic brain injury at 3 and 12 months post-injury: a prospective study. Brain Injury. (2009) 23:489–97. 10.1080/02699050902926309 19484622

[54] Skilbeck C Dean T Thomas M Slatyer M. Impaired National Adult Reading Test (NART) performance in traumatic brain injury. Neuropsychol Rehabil. (2013) 23:234–55. 10.1080/09602011.2012.747968 23245593

[55] Steward KA Gerstenecker A Triebel KL Kennedy R Novack TA Dreer LE . Twelve-month recovery of medical decision-making capacity following traumatic brain injury. Neurology. (2016) 87:1052–9. 10.1212/WNL.0000000000003079 27511180 PMC5027810

[56] Singh R Choudhri K Sinha S Mason S Lecky F Dawson J. Global outcome after traumatic brain injury in a prospective cohort. Clin Neurol Neurosurg. (2019) 186:105526. 10.1016/j.clineuro.2019.105526 31585337

[57] Sterr A Herron KA Hayward C Montaldi D. Are mild head injuries as mild as we think? neurobehavioral concomitants of chronic post-concussion syndrome. BMC Neurol. (2006) 6:7. 10.1186/1471-2377-6-7 16460567 PMC1382265

[58] Teasdale G Jennett B. Assessment of coma and impaired consciousness: a practical scale. The Lancet. (1974) 304:81–4. 10.1016/S0140-6736(74)91639-0 4136544

[59] Theadom A Starkey NJ Dowell T Hume PA Kahan M McPherson K . Sports-related brain injury in the general population: an epidemiological study. J Sci Med Sport. (2014) 17:591–6. 10.1016/j.jsams.2014.02.001 24602688

[60] Sport concussion assessment tool - 5th edition. Br J Sports Med. (2017) 51:851–8. 28446451 10.1136/bjsports-2017-097506SCAT5

[61] Greco T Ferguson L Giza C Prins ML. Mechanisms underlying vulnerabilities after repeat mild traumatic brain injuries. Exp Neurol. (2019) 317:206–13. 10.1016/j.expneurol.2019.01.012 30853388

[62] Guskiewicz KM McCrea M Marshall SW Cantu RC Randolph C Barr W . Cumulative effects associated with recurrent concussion in collegiate football players: the NCAA concussion study. JAMA. (2003) 290:2549–55. 10.1001/jama.290.19.2549 14625331

[63] Master CL Katz BP Arbogast KB McCrea MA McAllister TW Pasquina PF . Differences in sport-related concussion for female and male athletes in comparable collegiate sports: a study from the NCAA-DoD Concussion Assessment, Research and Education (CARE) Consortium. Br J Sports Med. (2021) 55:1387–94. 10.1136/bjsports-2020-103316 33355211

